#  Longitudinal Maintenance of Cognitive Health in Centenarians in the 100-plus Study

**DOI:** 10.1001/jamanetworkopen.2020.0094

**Published:** 2020-02-26

**Authors:** Nina Beker, Sietske A. M. Sikkes, Marc Hulsman, Niccolò Tesi, Sven J. van der Lee, Philip Scheltens, Henne Holstege

**Affiliations:** 1 Alzheimer Center Amsterdam, Department of Neurology, Amsterdam Neuroscience, Vrije Universiteit Amsterdam, Amsterdam UMC, Amsterdam, the Netherlands; 2 Department of Clinical Psychology, Neuropsychology, and Developmental Psychology, Faculty of Behavioural and Movement Sciences, Vrije Universiteit, Amsterdam, the Netherlands; 3 Department of Clinical Genetics, Amsterdam Neuroscience, Vrije Universiteit Amsterdam, Amsterdam UMC, Amsterdam, the Netherlands

## Abstract

**Importance:**

Some individuals who reach ages beyond 100 years in good cognitive health may be resilient against risk factors associated with cognitive decline. Exploring the processes underlying resilience may contribute to the development of therapeutic strategies that help to maintain cognitive health while aging.

**Objective:**

To identify individuals who escape cognitive decline until extreme ages and to investigate the prevalence of associated risk factors.

**Design, Setting, and Participants:**

The 100-plus Study is a prospective observational cohort study of community-based Dutch centenarians enrolled between 2013 and 2019 who were visited annually until death or until participation was no longer possible. The centenarians self-reported their cognitive health, as confirmed by a proxy. Of the 1023 centenarians approached for study inclusion, 340 fulfilled the study criteria and were included in analyses. Data analysis was performed from April 2019 to December 2019.

**Main Outcomes and Measures:**

Cognition was assessed using the Mini-Mental State Examination (MMSE). To identify centenarians who escape cognitive decline, this study investigated the association of baseline cognition with survivorship and cognitive trajectories for at least 2 years of follow-up using linear mixed models, adjusted for sex, age, and education. This study investigated the prevalence of apolipoprotein E (*APOE*) genotypes and cardiovascular disease as risk factors associated with cognitive decline.

**Results:**

At baseline, the median age of 340 centenarians was 100.5 years (range, 100.0-108.2 years); 245 participants (72.1%) were female. The maximum survival estimate plateaued at 82% per year (95% CI, 77% to 87%) across centenarians who scored 26 to 30 points on the baseline MMSE (hazard ratio, 0.56; 95% CI, 0.42 to 0.75; *P* < .001), suggesting that an MMSE score of 26 or higher is representative of both cognitive and physical health. Among the 79 centenarians who were followed up for 2 years or longer, those with baseline MMSE score less than 26 experienced a decline in MMSE score of 1.68 points per year (95% CI, −2.45 to −0.92 points per year; *P* = .02), whereas centenarians with MMSE scores of 26 or higher at baseline experienced a decline of 0.71 point per year (95% CI, −1.08 to −0.35 points per year). For 73% of the centenarians with baseline MMSE scores of 26 or higher, no cognitive changes were observed, which often extended to ensuing years or until death. It is estimated that this group is representative of less than 10% of Dutch centenarians. In this group, 18.6% carried at least 1 *APOE-ε4* allele, compared with 5.6% of the centenarians with lower and/or declining cognitive performance.

**Conclusions and Relevance:**

Most centenarians who scored 26 or higher on the MMSE at baseline maintained high levels of cognitive performance for at least 2 years, in some cases despite the presence of risk factors associated with cognitive decline. Investigation of this group might reveal the processes underlying resilience against risk factors associated with cognitive decline.

## Key Points

**Question:**

Is it possible to identify centenarians who escape cognitive decline?

**Findings:**

In this cohort study of 340 centenarians, most individuals who scored 26 or higher on the Mini-Mental State Examination at study inclusion maintained this level of performance for at least 2 years of follow-up, despite having risk factors associated with cognitive decline. This group represents less than 10% of the centenarians in the Dutch population.

**Meaning:**

Using the Mini-Mental State Examination, this study identified a small subgroup of centenarians who maintained high levels of cognitive functioning and showed resilience against risk factors associated with cognitive decline.

## Teaser

This cohort study of Dutch centenarians examines individuals who escape cognitive decline until extreme ages and investigates the prevalence of risk factors associated with cognitive decline.

## Introduction

Improvements in medical care and living conditions in the last century have led to an increased likelihood of reaching extreme ages. By 2100, an estimated 25 million people worldwide will have reached the age of 100 years.^1,2^ This increase in life expectancy will lead to a higher prevalence of age-related disorders such as dementia. Dementia incidence increases exponentially with age, to an estimated 40% per year in individuals aged 100 years.^3^ Results from cross-sectional studies indicated that approximately 50% of centenarians show clear symptoms of dementia and that 25% of centenarians exhibit at least some symptoms of cognitive impairment, whereas approximately 25% are considered to be cognitively healthy.^4,5,6,7^ However, these numbers vary across studies, because it is difficult to define cognitive health in centenarians, in part because of their physical frailty, which hampers accurate cognitive assessment.^4,7^

Despite the high risk of developing dementia at extreme ages, some centenarians escape developing symptoms of cognitive decline until well beyond age 100 years. Several studies^8,9,10,11^ have documented supercentenarians (ie, aged ≥110 years) who maintained high levels of cognitive functioning during their entire lifetime. In one of these studies,^11^ an extensive cognitive assessment of a supercentenarian indicated that she was free of symptoms associated with cognitive impairment at age 112 years, and follow-up assessments indicated that she remained cognitively healthy until her death at age 115 years. The existence of these extraordinary individuals shows that it is possible to maintain cognitive health throughout very late life or until death. Considering their advanced age, these individuals are potentially resilient against risk factors associated with cognitive decline.^12^

It is currently unclear to what extent individuals who reach age 100 years in good cognitive health are able to escape cognitive decline. Once one reaches age 100 years in good cognitive health, what are the chances of maintaining high levels of cognitive functioning beyond age 100 years or until death? What level of cognitive performance is indicative of maintained cognitive functioning? Answers to these questions allow for further exploration of the factors underlying cognitive resilience, which refers to the aptitude to maintain a given level of cognitive ability despite repeated insults.^12^ To investigate this, it is necessary to follow trajectories of cognitive change and to investigate the extent to which centenarians are exposed to risk factors associated with cognitive decline.

Longitudinal studies of centenarians are scarce because they are complicated by the high risk of frailty and mortality,^2,13,14,15,16,17,18^ which skews such studies toward investigation of a subgroup of individuals who survive longest: cognitively intact, physically healthy individuals.^13^ Here, we used the known association between cognitive performance and survival to identify centenarians who were able to uphold high levels of cognitive performance across an extended period.^19,20,21,22^ In a longitudinal assessment of centenarians from the Dutch 100-plus Study cohort, we first investigated the association between baseline cognition and expected survivorship, and then we investigated the association between baseline cognition and trajectories of cognitive and physical functioning across a period of at least 2 years after baseline. Finally, we investigated the prevalence of risk factors associated with cognitive decline in this cohort of centenarians.

## Methods

### Participants and Data Collection

**Figure 1.**
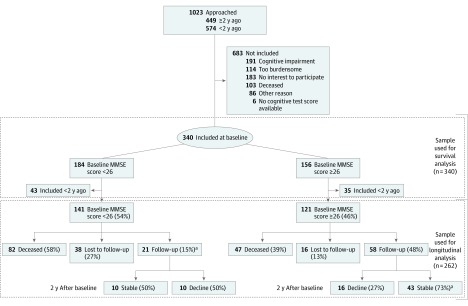
Flowchart of the Sample and Different Subgroups and Percentage of Subgroups of Centenarians Within Longitudinal Analysis MMSE indicates Mini-Mental State Examination. ^a^One centenarian scored less than 26 on the MMSE at baseline, but had a score greater than or equal to 26 after 2 years of follow-up.

We included participants from the 100-plus Study, a prospective cohort study of Dutch centenarians who self-reported being cognitively healthy, as confirmed by a proxy. Recruitment and inclusion procedures have been described elsewhere.^23^ We approached 1023 centenarians for study inclusion, of whom 340 agreed and were eligible to participate (Figure 1). Others were excluded because of cognitive impairment (191 centenarians), when participation was considered too burdensome (114 centenarians), or when there was no interest in participating (183 centenarians). To perform longitudinal analyses, we selected 79 centenarians who were visited for follow-up at least 2 years after baseline (1 centenarian missed the first follow-up visit, but did participate in the second follow-up visit). This sample was derived from 262 centenarians who were included in the study more than 2 years ago (Figure 1). Centenarians were visited at their home residence by trained researchers with neuropsychological and/or medical training. Visits were performed annually and included cognitive testing and questions regarding well-being and physical functioning. Centenarians were followed up until death, until frailty no longer permitted assessment, or until participants declined follow-up visits for other reasons. Visits took place between January 2013 and April 2019 (data freeze).

The Medical Ethics Committee of the Amsterdam UMC approved this study, and written informed consent was obtained from all participants. The study has been conducted in accordance with the declaration of Helsinki. This study follows the Strengthening the Reporting of Observational Studies in Epidemiology (STROBE) reporting guideline for observational studies.

### Measures

Global cognitive functioning was assessed at baseline and at follow-up visits using the Mini-Mental State Examination (MMSE), which has 11 items and a score range of 0 to 30, with higher scores indicating better performance.^24^ Physical functioning was evaluated using the Dutch Barthel Index (BI) at baseline and follow-up visits.^25^ When centenarians were community dwelling, an interview version of the BI was administered. When centenarians were care dependent, a proxy or a caregiver filled in the observation version of the BI. Scores ranged from 0 to 20, with scores of 15 or higher indicating physical independence.^26^

### Apolipoprotein E Genotyping

We genotyped or imputed apolipoprotein E (*APOE*) genotypes using standard procedures as previously described.^23^ Carrying 1 or 2 alleles of *APOE-ε4* was considered a risk-increasing factor associated with cognitive decline (*APOE*-ε3ε4, *APOE*-ε2ε4, or *APOE*-ε4ε4), whereas carrying 1 or 2 alleles of *APOE-ε2* (*APOE*-ε2ε2 or *APOE*-ε2ε3) was considered a protective factor against cognitive decline (note that the *APOE*-ε2ε4 genotype was considered a risk-increasing factor).^27,28,29,30^

### Baseline Clinical and Demographic Characteristics

We investigated baseline differences in age, sex, education, living situation, hearing, vision, mobility, use of prescription medications, and history of heart disease, stroke and/or transient ischemic attack, diabetes, and hypertension between subgroups of centenarians. We used the International Standard Classification of Education 1997 to determine the level of education.^31^ Centenarians who lived in a care center facility, in independent housing but with around-the-clock care available, or with family members were classified as living dependently; if not, they were classified as living independently. We classified hearing and vision capacities as good to moderate and as poor to very poor on the basis of the self-report of the centenarians and their family members and on the observations of the researcher during home visits.^23,32^ History of heart disease, stroke and/or transient ischemic attack, diabetes, and hypertension was based on the medical report from the centenarian’s general practitioner (GP) or, if not available, the self-report of the centenarians and/or a proxy. We determined medication use by the total number of prescription medication categories used by the centenarians according to the medication lists provided by their GPs (eg, cardiac medication, diuretics, anticoagulants, laxatives, antacids, analgesic, sedatives, antidiabetics, antidepressants, iron supplements, and others).

### Statistical Analysis

#### Sensitivity Analyses

A fraction of the centenarians declined a follow-up visit or could no longer be tested because of physical and/or cognitive impairments. To prevent an underrepresentation of those with cognitive and physical decline, we asked a proxy to fill in the BI and the short form of the Informant Questionnaire on Cognitive Decline in the Elderly (IQCODE) as an additional measure of cognitive functioning.^33^ The IQCODE consists of 16 items on a 5-point scale. For analyses, we used the mean score of all items, ranging from 1 to 5, with lower scores indicating less cognitive decline.

#### Missing Items

In some instances, decline in vision, hearing, or motor problems led to missing items. We corrected for missing items using mean imputation methods (BI and IQCODE) or multiple imputation methods (MMSE) if fewer than 20% of the items were missing.^23^ The MMSE scores were imputed for 93, 14, and 79 of the participants assessed at baseline (340 participants total), first follow-up visit (78 participants total), and second follow-up visit (79 participants total), respectively. The MMSE scores of 2 centenarians (1 each at baseline and the first follow-up visit) were excluded from analyses because more than 20% of the items were missing. Missing items on the descriptive characteristics were not imputed, such that analyses including these variables were based on smaller subsets (see Table 1 for an overview).

**Table 1.** Baseline Characteristics of the Total Sample and Participants Who Were Followed up for 2 Years or Longer

Characteristic	Total Participants Included at Baseline, No. (%) (N = 340)^a^	Participants Followed up for ≥2 y, No. (%)			*P* Value^b^
		Total (n = 79)	Maintained Cognitive Health (n = 43)	Did Not Maintain Cognitive Health (n = 36)	
Age, median (IQR), y	100.5 (100.2-101.7)	100.7 (100.2-101.7)	100.5 (100.2-101.6)	100.7 (100.2-101.6)	.71
Female	245 (72.1)	60 (75.9)	34 (79.1)	26 (72.2)	.66
Education level^c^					
Low	96 (28.2)	19 (24.1)	8 (18.6)	11 (30.6)	.15
Medium	192 (56.5)	50 (63.3)	27 (62.8)	23 (63.9)	
High	52 (15.3)	10 (12.7)	8 (18.6)	2 (5.6)	
Living situation					
Independent	190 (55.9)	50 (63.3)	30 (69.8)	20 (55.6)	.28
Dependent	150 (44.1)	29 (36.7)	13 (30.2)	16 (44.4)	
Mobility					
Able to walk independently^d^	253 (78.8)	74 (94.8)	42 (97.7)	32 (91.4)	.29
Able to walk with help of another person	18 (5.6)	2 (2.6)	0	2 (5.7)	
Able to move independently in a wheelchair	25 (7.8)	2 (2.6)	1 (2.3)	1 (2.9)	
Not able to move independently in a wheelchair	25 (7.8)	0	0	0	
Vision					
Good to moderate	259 (78.0)	67 (85.9)	37 (88.1)	30 (83.3)	.78
Poor to very poor	73 (22.0)	11 (14.1)	5 (11.9)	6 (16.7)	
Hearing					
Good to moderate	292 (86.9)	75 (94.9)	41 (95.3)	34 (94.4)	>.99
Poor to very poor	44 (13.1)	4 (5.1)	2 (4.7)	2 (5.6)	
Medication categories^e^					
Participants, No.	324	76	42	34	.79
Categories, No., median (IQR)	3.0 (2.0-5.0)	3.0 (1.0-4.0)	3.0 (1.0-4.0)	3.0 (1.0-4.0)	
Mini-Mental State Examination score, median (IQR)	25.0 (22.0-27.5)	27.0 (25.5-29.0)	28.0 (27.0-29.0)	25.0 (22.8-26.7)	<.001
Barthel index					.008
Participants, No.	304	73	39	34	
Score, median (IQR)	16.0 (12.0-19.0)	17.0 (14.0-19.0)	18.0 (15.5-20.0)	15.3 (13.3-18.0)	
Allele carriers, participants, No./total No. (%)					
*APOE*-ε4	48/295 (16.3)	10/79 (12.7)	8/43 (18.6)^f^	2/36 (5.6)	.16
*APOE*-ε2	68/295 (23.1)	17/79 (21.5)	9/43 (20.9)	8/36 (22.2)	.93
Medical history, participants, No./total No. (%)					
Heart disease^g^	207/298 (69.5)	48/78 (61.5)	27/42 (64.3)	21/36 (58.3)	.76
Diabetes	21/286 (7.3)	5/78 (6.4)	4/42 (9.5)	1/36 (2.8)	.45
Stroke and/or transient ischemic attack	94/312 (30.1)	21/79 (26.6)	13/43 (30.2)	8/36 (22.2)	.58
Hypertension	195/311 (62.7)	45 (57.0)	26 (60.5)	19 (52.8)	.65

Abbreviations: *APOE*, apolipoprotein E gene; IQR, interquartile range.

^a^ Total number was reported in columns separately in case of missing data.

^b^ Comparison between 43 centenarians who maintained cognitive health (Mini-Mental State Examination score ≥26) after 2 years and the remaining 36 centenarians.

^c^ Education level was determined according to the International Standard Classification of Education 1997^31^ and was divided into low (primary education level or lower), medium (secondary education level or higher), and high (tertiary education level or higher).

^d^ With or without the use of a walking stick or walker.

^e^ Prescription medication categories represents the total number of categories for which centenarians used at least 1 medication.

^f^ One centenarian was homozygous for the *APOE-ε4* allele.

^g^ Heart disease included 1 of the following: valvular heart disease, having a pacemaker, thrombosis, angina pectoris, congestive heart failure, irregular heartbeat, myocardial infarction, and dyslipidemia.

#### Survival

Information on survival until April 2019 was obtained via family report. Participants who were alive were censored at the last visit or when last confirmed alive by family members or caregivers. After having been informed about the death of a centenarian, medical reports from the GP were obtained to acquire information about cognitive health shortly before death. Yearly survival estimates and 95% CIs were calculated by taking the square root of the Kaplan-Meier survival estimates over 2-year survival (survival percentage equals the yearly survival percentage raised to the power of *n* years).

#### Association Between Cognitive Functioning and Survival

To investigate the association between baseline cognition and expected survivorship per year, we plotted yearly survival estimates per MMSE score range, using a sliding window with a size of 3 MMSE points (ie, <20, 20-22, 21-23, …, 28-30). We stratified centenarians according to the MMSE score range associated with the highest yearly survival estimates. This subdivision was statistically validated using Cox proportional hazard models and is shown as hazard ratios with 95% CIs. We investigated differences in baseline demographic and clinical characteristics between groups using χ^2^ tests for categorical variables and *t* tests or Kruskal-Wallis tests (in case of nonnormality) for continuous variables.

#### Trajectories of Cognitive and Physical Functioning

We used linear mixed models to explore the trajectories of MMSE or BI scores over time for centenarians for whom at least 2 follow-up visits were available. Time (in years) was used as a continuous independent variable, and MMSE or BI scores were used as continuous dependent variables. All models included a random intercept, and random slopes were explored. To stratify groups according to decline in MMSE scores over time, we calculated a reliable change index.^34^ Sex, age, and education were included as covariates in the models.

Statistical analyses were performed with R statistical software version 3.3.3 (R Project for Statistical Computing) with survminer, KMsurv, survival, lme4, and car packages. Two-sided *P *<* *.05 was considered statistically significant. Data analysis was performed from April 2019 to December 2019.

## Results

### Baseline Characteristics

At baseline, the 340 included centenarians (245 [72.1%] female) had a median age of 100.5 years (range, 100.0-108.2 years) and had been born between 1906 and 1919. Most (56.5%) had a medium education level. Of the total sample, 55.9% were community dwelling and 78.8% were able to walk with or without the use of a walking stick or walker. The large majority had good-to-moderate vision (78.0%) and hearing (86.9%) abilities. The centenarians scored a median (interquartile range [IQR]) of 25.0 (22.0-27.5) points on the MMSE at baseline, and a median (IQR) of 16.0 (12.0-19.0) points on the BI (Table 1).

### Maximum Survival in Centenarians Who Scored 26 Points or Higher on the Baseline MMSE

**Figure 2.**
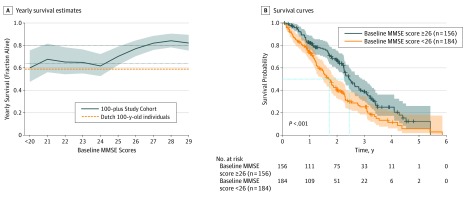
Association of Mini-Mental State Examination (MMSE) Scores With Survival A, Graph shows yearly survival estimates for the 100-plus Study cohort. Dashed orange line shows the survival fraction of the individuals aged 100 years within the Dutch general population, which was derived from the Netherlands Central Bureau of Statistics.^35^ Dotted lines show mean survival percentage of centenarians with baseline MMSE score less than 24 (bottom) and mean survival percentage of centenarians with baseline MMSE score less than 26 (top). Solid line and shaded area show mean (95% CI) survival for participants in the 100-plus Study cohort. The MMSE scores were associated with survival using sliding windows with a size of 3 MMSE points. B, Graph shows Kaplan-Meier survival curves of centenarians with high (≥26 [gray]) and low (<26 [orange]) MMSE scores at baseline.

After censoring, the survival percentage of all centenarians who scored 26 to 30 points on the baseline MMSE plateaued at a mean of 82% per year (95% CI, 77%-87%). We observed that the expected survival gradually decreased in centenarians with a baseline MMSE score below 26 and that it was lowest (<64%) in centenarians who scored less than 24 on the baseline MMSE (95% CI, 56%-72%). The survival of the centenarians who scored less than 24 on the MMSE was comparable with the survival of individuals aged 100 years in the overall Dutch population,^35^ of whom the majority are estimated to have dementia or cognitive impairment (Figure 2A). The longer survival for the centenarians who scored 26 or higher on the baseline MMSE was confirmed by Cox regression analyses, showing a 44% hazard reduction for mortality compared with those who scored less than 26 (hazard ratio for MMSE score ≥26 adjusted for age, sex, and education, 0.56; 95% CI, 0.42-0.75; *P* < .001) (Figure 2B).

### Trajectories of Cognitive Functioning: Identification of Centenarians With Stable Cognitive Performance

Of the 79 centenarians who could be followed up for 2 years, the first follow-up visit occurred a median (IQR) of 1.01 (0.99-1.03) years after baseline, and the second follow-up visit occurred a median (IQR) of 2.05 (1.99-2.09) years after baseline. The observed maximum survivorship in those who scored between 26 and 30 points led us to stratify centenarians according to whether they scored 26 or higher or less than 26 on the MMSE at baseline.

**Figure 3.**
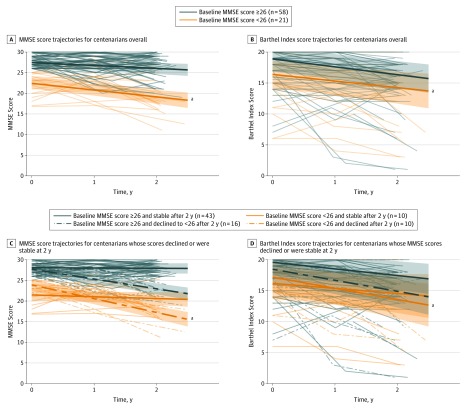
Trajectories of Mini-Mental State Examination (MMSE) and Barthel Index Scores Linear mixed-models fixed effects of MMSE score (range, 0-30) and Barthel Index scores (range, 0-20) over 2 years stratified for subgroups are shown according to baseline MMSE (≥26 vs <26) and whether the centenarian’s scores declined or remained stable. A, MMSE trajectories of centenarians with baseline MMSE scores 26 or higher and less than 26. B, Barthel Index trajectories of centenarians with baseline MMSE scores 26 or higher and less than 26. (Note that the upper limit of the 95% CI is clipped at the maximum score of 20.) C, MMSE trajectories of centenarians with baseline MMSE scores 26 or higher and less than 26 at baseline and who declined or were stable on the MMSE after 2 years. D, Barthel Index trajectories of centenarians with baseline MMSE scores 26 or higher and less than 26 at baseline and who declined or were stable on the MMSE after 2 years. (Note that the upper limit of the 95% CI is clipped at the maximum score of 20.) ^a^Centenarians who scored less than 26 on the MMSE at baseline. Because of the high level of frailty and mortality in this group, many of them could not be followed up for 2 years. Hence, the decline in MMSE and Barthel Index observed in the models for this specific subgroup possibly represents an underestimation of the cognitive and physical decline.

Of the 79 centenarians, 58 (73%) scored 26 points or higher on the MMSE at baseline and their scores declined by a mean of −0.71 point per year (95% CI, −1.08 to −0.35 points per year) across 2 years. In contrast, the scores of the 21 centenarians (27%) who scored less than 26 points on the MMSE at baseline declined significantly faster, by a mean of −1.68 points per year (95% CI, −2.45 to −0.92 points per year) across 2 years (β for MMSE score ≥26 × time, 0.93; 95% CI, 0.17 to 1.68; *P* = .02) (Table 2; Figure 1 and Figure 3A). One centenarian scored 25 on the MMSE at baseline but scored 26 points or higher during the first and second follow-up visits; as a result, this centenarian was transferred to the group with baseline MMSE scores of 26 or higher for downstream analyses (Figure 1).

**Table 2.** Separate Linear Mixed Models Examining Change in MMSE and Barthel Index Scores Over 2 Years Stratified by Groups^a^

Model	Total		Stable		Decline	
	β (95% CI)	*P* Value	β (95% CI)	*P* Value	β (95% CI)	*P* Value
MMSE score ≥26						
Participants, No.	58		43^b^		16	
MMSE model						
Time	−0.71 (−1.08 to −0.35)	<.001	−0.07 (−0.32 to 0.18)	.57	−2.39 (−3.05 to −1.73)	<.001
*R*^2^ (*M*/*C*)	0.10/0.59		0.06/0.34		0.59/0.59	
Barthel Index model						
Time	−1.28 (−1.76 to −0.80)	<.001	−1.06 (−1.51 to −0.62)	<.001	−1.79 (−3.04 to −0.53)	.01
*R*^2^ (*M*/*C*)	0.13/0.81		0.18/0.83		0.12/0.75	
MMSE score <26						
Participants, No.	21		10		10	
MMSE model						
Time	−1.68 (−2.45 to −0.92)	<.001	−0.44 (−1.24 to 0.37)	.30	−3.35 (−4.18 to −2.52)	<.001
*R*^2^ (*M*/*C*)	0.24/0.55		0.12/0.59		0.76/0.76	
Barthel Index model						
Time	−1.31 (−2.11 to −0.52)	.004	−0.81 (−1.74 to 0.11)	.12	−1.88 (−3.01 to −0.75)	.01
*R*^2^ (*M*/*C*)	0.20/0.83		0.37/0.95		0.27/0.61	

Abbreviation: MMSE, Mini-Mental State Examination.

^a^ Models are performed separately for MMSE and Barthel Index and include time in years as fixed factors and independent variables and MMSE or Barthel Index scores as dependent variables. Age, sex, and education were included as covariates. All models include random intercept and slopes. *C* (conditional *R*^2^) is defined as the proportion of variance explained by fixed and random factors. *M* (marginal *R*^2^) is defined as the proportion of variance explained by fixed factors. For the entire cohort of 79 participants who were followed up for 2 years or longer, for the MMSE model with time as the variable, β = −0.97 (95% CI, −1.31 to −0.63) and *R*^2^ (*M*/*C*) = 0.06/0.76 (*P* < .001) and for the Barthel Index model, β = −1.28 (95% CI, −1.69 to −0.87) for time and *R*^2^ (*M*/*C*) = 0.13/0.83 (*P* < .001).

^b^ One centenarian scored 25 on the MMSE at baseline, and then scored 26 or more points during the first and second follow-up visits; this centenarian was transferred to the stable group of centenarians scoring 26 points or higher on the MMSE.

Of the 58 centenarians with a baseline MMSE score of 26 or higher, a subgroup of 43 centenarians (73%) retained a score of 26 points or higher on the MMSE. Their MMSE scores declined by a mean of −0.07 point per year during the 2 years of follow-up (95% CI, −0.32 to 0.18 point per year; *P* = .57). The baseline MMSE score of these centenarians was uniformly distributed between 26 and 30 (median [IQR] baseline MMSE score, 28 [27-29] points). Of these 43 centenarians, 25 (58%) had died, and for 10 individuals the cognitive status at the time of death was known from GP reports. The GPs subjectively reported that 7 centenarians (70%) had retained good cognitive health until the day of their death. For 14 of the 43 cognitively stable centenarians, at least 3 years of follow-up data were available, and the cognitive performance of 10 centenarians (71%) remained stable with an MMSE score of 26 points or higher. Of the 4 centenarians who were available for follow-up after 4 years, all 4 (100%) had retained an MMSE score of 26 or higher. The group of high-performing, cognitively stable centenarians represents 16.4% of the centenarians included in the 100-plus Study 2 or more years before data freeze (262 participants), and 9.6% of all centenarians approached for study inclusion (449 participants) (Figure 1).

Of the 16 centenarians who scored 26 or higher on the baseline MMSE but whose MMSE scores declined to less than 26 during follow-up (26%), we observed a decrease of a mean of −2.39 points per year (95% CI, −3.05 to −1.73 points per year) across 2 years (Table 2 and Figure 3C). Of the 20 centenarians who scored less than 26 on the MMSE at baseline, 10 remained cognitively stable for 2 years despite their low MMSE score (50%), according to the reliable change index (>1.85 points decline per year, during 2 years). The MMSE score of these individuals declined by a mean of −0.44 point per year (95% CI, −1.24 to 0.37 points per year), whereas the MMSE score of the 10 remaining centenarians declined by a mean of −3.35 points per year (95% CI, −4.18 to −2.52 points per year) (Table 2 and Figure 3C).

### Physical Functioning and Trajectories

The 58 centenarians who scored 26 or higher on the MMSE had similar rates of decline on the BI (β, −1.28; 95% CI, −1.76 to −0.80; *P* < .001) compared with the 21 centenarians who scored less than 26 at baseline (β, −1.31; 95% CI, −2.11 to −0.52; *P* = .004) (Table 2 and Figure 3B). When we considered the 43 cognitively stable centenarians, we found that this subgroup remained stable across several aspects of physical functioning: 85% were physically independent at baseline (BI score ≥15), which changed to 67% after 2 years of follow-up; 98% and 95% were independently walking at baseline and after 2 years, respectively; and 70% and 60% were independently living at baseline and after 2 years, respectively. Nevertheless, we did observe a significant decline in BI scores in these 43 cognitively stable centenarians (β, −1.06; 95% CI, −1.51 to −0.62; *P* < .001), but this was less than the physical decline observed in the 16 centenarians who cognitively declined from MMSE scores of 26 or higher to scores of less than 26 in 2 years (β, −1.79; 95% CI, −3.04 to −0.53; *P* = .01), but the difference did not reach statistical significance (*P* = .19).

Although the 43 cognitively stable centenarians had a higher BI score at baseline compared with all 36 other centenarians (median [IQR], 18.0 [15.5-20.0] points vs 15.3 [13.3-18.0] points; *P* = .008), we did not find differences in the prevalence of diabetes (4 [9.5%] vs 1 [2.8%]), medication use (median [IQR] medication categories, 3.0 [1.0-4.0] for both groups), and other clinical and demographic variables (Table 1). Moreover, we found no differences between the 43 centenarians who maintained an MMSE score of 26 or higher during at least 2 years (“maintainers”) and the 36 centenarians who scored less than 26 on the MMSE at baseline and/or after 2 years of follow-up (“decliners”) in terms of heart disease (27 [64.3%] vs 21 [58.3%]), stroke and/or transient ischemic attack (13 [30.2%] vs 8 [22.2%]), and hypertension (26 [60.5%] vs 19 [52.8%]) (Table 1).

### Analysis of the *APOE* Genotype as a Risk Factor Associated With Cognitive Decline

Of the whole group of centenarians, 23.1% carried an *APOE-ε2* protective allele, compared with 12.4% of the overall Dutch middle-aged population,^23^ which reiterates our previous observation that centenarians are, on average, 2-fold more likely than the general population to carry an *APOE-ε2* allele. In the current study, we found that of the 43 maintainers, 9 (20.9%) carried the protective *APOE-ε2* allele (9 *APOE*-ε2ε3) compared with a similar 8 (22.2%) of the 36 decliners (1 *APOE*-ε2ε2 and 7 *APOE*-ε2ε3) (Table 1). In contrast, of the 340 centenarians included at baseline, 16.3% carried a risk-increasing *APOE-ε4* allele, compared with 27.2% of the overall Dutch middle-aged population,^23^ reiterating our previous observation that centenarians are, on average, approximately 2-fold less likely than the general population to carry an *APOE-ε4* allele. Specifically, of the 43 maintainers, 8 (18.6%) carried an *APOE-ε4* allele, of whom 1 was homozygous (5 *APOE*-ε3ε4, 2 *APOE*-ε2ɛ4, and 1 *APOE*-ε4ε4), whereas of the 36 decliners, only 2 (5.6%) carried an *APOE-ε4* allele (2 *APOE*-ε3ε4). This difference did not reach statistical significance, possibly because of the low sample size (Table 1).

### Sensitivity Analyses for Study Attrition

Selective study attrition might differentially influence the physical and cognitive trajectories between groups. We performed a sensitivity analysis to account for the 54 centenarians who were still alive 2 years after baseline inclusion but who could no longer be visited because of overall symptoms of frailty: 38 (70%) of these centenarians scored less than 26 points on the baseline MMSE. The 77 centenarians who were still available for follow-up visits (BI was unknown for 2 centenarians) scored significantly higher on the BI compared with the 13 centenarians for whom a proxy filled out the BI after 2 years (mean [SD] BI score, 13.8 [4.6] points vs 8.2 [5.7] points; *P* = .005). Also, the rate of decline in BI was lower in the group that was still available for follow-up visits, although this difference did not reach statistical significance (mean [SD] change in BI score between baseline and the second follow-up visit, 2.6 [3.7] points vs 5 [4.7] points; *P* = .12). This was confirmed by the finding that for 10 of the 54 centenarians who were no longer available for follow-up visits, a proxy had filled in the IQCODE; they had declined significantly more (ie, higher IQCODE score) compared with 67 centenarians who were still fit enough for a follow-up visit and for whom IQCODE scores were available (mean [SD] IQCODE score, 4.3 [0.6] points vs 3.5 [0.5] points; *P* = .002).

## Discussion

Previous studies^5,36,37,38^ showed that it is possible to reach extreme ages without developing symptoms of cognitive impairment. However, it is thus far unclear what level of cognitive performance is associated with the ability to maintain cognitive health and which fraction of those who reach age 100 years in good cognitive health are able to maintain high levels of cognitive functioning beyond 100 years. Furthermore, the processes underlying the escape of cognitive decline are largely unknown. By making use of the known association between cognitive performance and survival, we found that a score of 26 or higher on the MMSE is representative of optimal cognitive and physical health status in centenarians. Next, we found that 73% of those who score above this MMSE threshold were able to maintain cognitive health for at least 2 years, which in most cases, extended to ensuing years or until death. These findings suggest that this subgroup escaped cognitive decline until extreme ages. We estimated that centenarians who maintained these high levels of cognitive functioning represent less than 10% of all centenarians in the Dutch population.

Our finding that most (73%) of the higher cognitive performers maintained their cognitive health for at least 2 years is in large agreement with findings in a smaller sample from the Heidelberg Centenarian Study,^39^ in which 71% (10 of 14) of the highest cognitive performers revealed no or only very little cognitive decline for 1.5 years. The MMSE scores of centenarians whose cognitive performance was maintained during this time were also at the higher end of the spectrum (the Heidelberg study used an abbreviated version of the MMSE, which prevents a direct comparison with the MMSE selection of a score of ≥26 points).

In the current study, the centenarians who maintained cognitive health had similar sensory and mobility capacities and use of medications, but had better overall physical health status at baseline and lower rates of physical decline compared with all other centenarians. However, our study indicates that despite their cognitive health, these centenarians were still vulnerable to overall physical decline. Indeed, age-associated diseases, including cardiovascular conditions, were previously found to be prevalent in our cohort.^23^ When considering cardiovascular diseases as a risk factor associated with cognitive decline, this offers an opportunity to identify whether centenarians who maintain good cognitive health are resilient against such risk factors by comparing their cardiovascular disease prevalence with the prevalence of the centenarians with a lower and/or declining cognitive performance.^40,41^ We found that the centenarians who maintained high levels of cognitive functioning did not have a lower prevalence of these diseases compared with all other centenarians in the cohort, which suggests that the escape of cognitive decline occurs irrespective of the effects of cardiovascular diseases.

Other well-known risk factors are the *APOE-ε2* and *APOE-ε4* alleles, which are known to have protective and risk-increasing effects, respectively, on cognitive decline and dementia.^27,28,29^ The prevalence of the protective *APOE-ε2* allele was similar in centenarians who maintained high levels of cognitive functioning and those with lower and/or declined cognitive performance. However, counterintuitively, the centenarians with lower and/or declined cognitive performance were less likely to have the *APOE-ε4* allele compared with the centenarians who maintained high levels of cognitive functioning, suggesting that this latter group might be resilient to the risk-increasing effect of the *APOE-ε4* allele on cognitive decline.^12,42^ Such resilience may be explained by, for example, an enrichment of protective genetic factors that counter the risk-increasing effect exerted by the *APOE-ε4* allele. Indeed, we previously found that the centenarians in the 100-plus Study had more protective genetic factors and fewer risk-increasing genetic factors associated with Alzheimer disease, compared with the population-matched sample of middle-aged individuals.^43^ One such protective genetic factor is a rare variant in the phospholipase C gamma–2 (*PLCG2*) gene, which is associated with the immune response and is associated with a notable 2-fold reduced risk of Alzheimer disease, Lewy body disease, and frontotemporal dementia.^44^ As an example of a person at very high risk of cognitive decline, we mention 1 centenarian in particular who, as of January 2020, was still alive at age 104 years, still cognitively healthy, and homozygous for the *APOE-ε4* risk allele.^44^ This centenarian carries the rare genetic variant in *PLCG2*, which may explain her resilience against *APOE-ε4* homozygosity. A similar finding of resilience was noted in a recent study^45^ that describes an individual who was homozygous for a rare protective variant in the *APOE-ε3* allele (Christchurch mutation), which is thought to explain the resilience against the effect of a mutation in the presenilin 1 gene, which is associated with autosomal dominant early-onset Alzheimer disease. However, we did not observe the rare protective Christchurch mutation in any of the centenarians in the present cohort, nor did we observe the rare protective variant in the *PLCG2* gene in all carriers of the *APOE-ε4* allele. Together, this suggests that next to these genetic protective factors, additional protective factors exist that confer resilience against risk factors associated with cognitive decline.

Our results on the prevalence of the *APOE-ε4* allele are in line with those of a study^46^ in which individuals aged 80 years were cognitively stable over 5 years despite their advanced age and one-fifth of them being *APOE-ε4* carriers. Moreover, the finding that one-half of these individuals aged 80 years were also found to be amyloid positive indicates that they might also be resilient against the neuropathological hallmarks associated with Alzheimer disease. Indeed, with increasing age, neuropathological changes become more common in cognitively healthy individuals also, suggesting that those who survive to higher ages may be increasingly selected to be resilient against the outcomes associated with such neuropathological changes.^47,48,49,50,51^ In a subsample of 40 centenarians of the 100-plus Study,^52^ we previously found that the levels of amyloid-β hyperphosphorylated tau and the levels of other neuropathological changes associated with neurodegenerative diseases varied widely among centenarians. This variability in neuropathological changes suggests that the processes underlying the resilience against the accumulation of neuropathological changes may be unique for each centenarian. Of note, because most of the centenarians who maintained high levels of cognitive functioning are currently alive or died only recently (as of this writing), these findings were based primarily on the centenarians who were included in our cohort of self-reported cognitively healthy centenarians, but who had lower cognitive performance. Future investigation of the neuropathological status in those who escaped cognitive decline may provide further insights into factors of resilience against neuropathological changes associated with neurodegenerative diseases.

### Limitations and Strengths

Several limitations must be taken into account when interpreting the results of this study. First, given the inclusion criteria of our study, we approached centenarians who are likely to be cognitively intact, such that our sample is not representative of the overall Dutch centenarian population. Therefore, the fraction of centenarians who were identified on the basis of maintained cognitive health, which was less than 10% of all those approached for study inclusion, is expected to be an overrepresentation of the fraction among all Dutch centenarians.

Furthermore, we recognize that the MMSE might be controversial as an instrument to evaluate cognitive health in centenarians, because its psychometric value was previously described as limited^53^ and it has been found to underestimate cognitive ability of centenarians who tend to have sensory deficits.^4,54,55^ We contended with this by imputing scores for missing items due to sensory problems. Furthermore, because aging is accompanied by susceptibility for decline in specific cognitive domains, it is necessary to take additional neuropsychological tests into account for a full characterization of cognitive health in centenarians in future studies.^56,57,58^ Interestingly, reports of MMSE thresholds for expected survival at (much) younger ages suggests that performance on the MMSE as a factor associated with expected survival remains at a score of approximately 25 to 26 points across ages, and it is therefore not age dependent.^59,60^ Of note, the MMSE threshold of 26 or higher for extended survival and the ability to maintain cognitive health should not be confused with the MMSE cutoff of 21 or lower that was previously found to be indicative of dementia in individuals older than 97 years.^61^

In addition, participant dropout is a common limitation in longitudinal studies, and centenarian studies are much affected by this because of the high risk of mortality and frailty.^2,13,14,15,16,17,18^ In the current study, we used the association between cognitive performance and survival to our advantage and determined that the MSSE scores of the longest survivors was 26 or higher. However, as a consequence, the centenarians who were no longer available for follow-up because of frailty or cognitive impairment concentrated among those who scored less than 26 on the MMSE at baseline, indicating that study attrition bias was especially observable in the lower-performing centenarians. This prevented the investigation of trajectories of cognitive status in the majority of the centenarians in this group, which suggests that both the fraction of decliners and the rate of decline in this group are an underestimation. To accommodate this, we performed a sensitivity analysis for the centenarians who dropped out of the study within the 2 years of follow-up. Indeed, the by-proxy analysis indicated more cognitive and physical decline in those who missed a follow-up visit, compared with those who were available for follow-up assessments.

For the aforementioned reasons, most studies of the oldest-old population focus on exceptional cognitive abilities using cross-sectional data.^46,62^ Together, the prospective design of our study, our approach using the known association between survival and cognition to our advantage, and our approach of contending with a survival and attrition bias, allowed us to examine trajectories of decline in this group of centenarians.^63^

## Conclusions

By use of an MMSE score threshold of 26 or higher, we identified a physically healthy subgroup of centenarians who maintained high levels of cognitive functioning for at least 2 years, which in most cases extended to ensuing years or until death. This group of centenarians escaped cognitive decline despite being exposed to associated risk factors, which suggests that they are resilient against these factors. The identification of cognitive resilience in centenarians indicates that they may have factors associated with successful aging. The further investigation of genetic and downstream molecular constellations in blood and brain tissues from these centenarians may lead to novel insights in processes that maintain cognitive health during extreme longevity.
